# Genetic diversity of *Elaeis oleifera* (HBK) Cortes populations using cross species SSRs: implication’s for germplasm utilization and conservation

**DOI:** 10.1186/s12863-017-0505-7

**Published:** 2017-04-19

**Authors:** Maizura Ithnin, Chee-Keng Teh, Wickneswari Ratnam

**Affiliations:** 1 Malaysian Palm Oil Board (MPOB), P.O.Box 10620, 50720 Kuala Lumpur, Malaysia; 2 Present Address: Biotechnology & Breeding Department, Sime Darby Plantation R&D Centre, 43400 Selangor, Malaysia; 3 School of Environmental and Natural Resource Sciences, Faculty of Science and Technology, Universiti Kebangsaan Malaysia, 43600 Bangi, Selangor Malaysia

**Keywords:** *Elaeis oleifera*, Microsatellites, Population structure, Germplasm utilization, Germplasm conservation

## Abstract

**Background:**

The *Elaeis oleifera* genetic materials were assembled from its center of diversity in South and Central America. These materials are currently being preserved in Malaysia as *ex situ* living collections. Maintaining such collections is expensive and requires sizable land. Information on the genetic diversity of these collections can help achieve efficient conservation via maintenance of core collection. For this purpose, we have applied fourteen unlinked microsatellite markers to evaluate 532 *E. oleifera* palms representing 19 populations distributed across Honduras, Costa Rica, Panama and Colombia.

**Results:**

In general, the genetic diversity decreased from Costa Rica towards the north (Honduras) and south-east (Colombia). Principle coordinate analysis (PCoA) showed a single cluster indicating low divergence among palms. The phylogenetic tree and STRUCTURE analysis revealed clusters based on country of origin, indicating considerable gene flow among populations within countries. Based on the values of the genetic diversity parameters, some genetically diverse populations could be identified. Further, a total of 34 individual palms that collectively captured maximum allelic diversity with reduced redundancy were also identified. High pairwise genetic differentiation (Fst > 0.250) among populations was evident, particularly between the Colombian populations and those from Honduras, Panama and Costa Rica. Crossing selected palms from highly differentiated populations could generate off-springs that retain more genetic diversity.

**Conclusion:**

The results attained are useful for selecting palms and populations for core collection. The selected materials can also be included into crossing scheme to generate offsprings that capture greater genetic diversity for selection gain in the future.

**Electronic supplementary material:**

The online version of this article (doi:10.1186/s12863-017-0505-7) contains supplementary material, which is available to authorized users.

## Background

Under Aracaceae, genus *Elaeis* consists of two important species, namely *E. guineensis* Jacq. and *E. oleifera* (HBK) Cortes. Both species yield oil used for food and non-food applications. In terms of their habitats, *E. guineensis* is endemic to the tropical lowlands of West and Central Africa, spreading from 16°N in Senegal to 15°S in Angola [1], whereas *E. oleifera* originates from Central and South America with natural distribution from 15°N in Honduras to less than 10°S in the north of Porto Velho in Brazil [2]. The natural American palms grow well at the river margins, semi-swamps and generally in open habitats [3]. The African origin*, E. guineensis* hybridizes with the American origin to produce F1 OxG hybrids [4].

Malaysian Palm Oil Board (MPOB), formally known as Palm Oil Research Institute of Malaysia (PORIM) has collected *E. oleifera* germplasm from Colombia, Panama, Costa Rica, Nicaragua and Honduras between 1981 and 1982. The collections were carried out on a collaborative basis, as explained in the Ethics section. Unlike the *E. guineensis*, the natural distribution of *E. oleifera* was very sparse [5]. Within a collection site, fewer palms were found thus, only few mature bunches were sampled. Between one and six bunches were harvested per site. In total, 167 bunches were collected from 59 sites distributed across the five countries. Half of seeds from each bunch were presented to the host country while the other half was brought to Malaysia. The seeds then were sowed and field planted in a Completely Randomized Design (CRD) and now serves as the *ex situ* living collection of *E. oleifera*.

Field evaluation of the *E. oleifera* genetic materials collected by MPOB revealed lower height increment as compared to the *E. guineensis* [6]. As low as 4.6 cm yr^-1^ height increment were recorded among selected *E. oleifera* palms particularly from Colombia, which is about one-tenth of that reported in *E. guineensis* [6]. Oil from *E. oleifera* palms contained higher level of unsaturated fatty acids, carotenes, vitamin E and sterol contents [7]. Selected palms collected from Costa Rica and Panama contained more than 3,000 ppm of carotene content [8], whereas those from *E. guineensis* possessed only between 400 and 1,000 ppm of carotene [9]. Palms from Colombia, Panama and Costa Rica exhibited low palmitic acid (15.8–18.6%) and high oleic acid (56.5–61.5%) contents [6], resulting in higher level of unsaturation in their oil, a value-added feature that can enhance palm oil marketing in temperate countries. In terms of bunch components, palms from Panama recorded highest mean fruit weight. For mesocarp content, fruits harvested from palms collected from Costa Rica showed the highest value, followed by Panama and Honduras [10]. Despite these, the American oil palm does not attract much interest among planters due to its extremely low oil yield (0.5 t ha^-1^yr^-1^) compared to the African oil palm that produce an average of 3–4 t ha^-1^yr^-1^ [10]. Thus, direct commercialization of the former is not possible. Nevertheless, selected oil palm agencies worldwide have incorporated *E. oleifera* into their breeding programs [11, 12] via backcross breeding scheme. In South American countries, where oil palm disease caused by bud rot commonly occurs, interspecific hybrids of both species have been widely adopted. It is believed that the interspecific hybrids are relatively more tolerant to this disease than the African oil palms.

Maintaining *ex situ* living collection of perennial species like *E. oleifera* requires huge financial support and land. One round of oil palm conventional breeding cycle takes 10 years. Typically, development of new and improved oil palm varieties needs 30–40 years. Therefore, the selected palms carrying genes for interesting traits identified from the germplasm collection must be appropriately preserved to ensure continuous access, in accordance with the long-term oil palm improvement program. In addition, the present *ex situ* living germplasm is exposed to diseases and climate change. Valuable palms may be lost at any time. Thus, an effective conservation program should be in place to preserve palms that carry genes of economically important traits as well as those that represent the diversity of the species for exploitation and selection gain in the future. The principle of core collection [13] where, optimum diversity is preserved within minimum redundancy and population size offers an efficient method for conserving the *E. oleifera* germplasm.

Unlinked microsatellite markers developed from *E. guineensis* are available publicly at http://tropgenedb.cirad.fr/tropgene/JSP/interface.jsp?module=OILPALM [14]. These SSRs have successfully been used for assessment of genetic diversity of *E. guineensis* [15], fingerprinting and construction of genetic linkage map for *E. guineensis* [16] and their interspecific hybrids [17, 18]. We initiated an effort to evaluate the genetic diversity of selected *E. oleifera* populations using *E. guineensis* SSR markers [14]. Our analysis included genetic materials assembled from the species’ natural distribution in selected countries in Central and South America. This work provides an overview of the genetic variation, structure and relatedness among individual and populations of the species that may help breeders and germplasm managers prioritize individual palms and populations for establishment of core collection.

## Results

### Transferability of *E. guineensis* microsatellite markers to *E. oleifera*

Of the 18 SSRs tested, 14 (77.78%) revealed scorable amplification products in the screening panel and were then genotyped across the entire samples set in the study. The amplified products observed in the *oleifera* samples were within the expected size as reported in [14] (Table 1). The four SSRs that failed to generate scorable profiles were mEgCIR1753, mEgCIR3785, mEgCIR3300 and mEgCIR3574.

**Table 1:** Informative SSRs used in genotyping the 532 accessions

No	Linkage group	Locus	Range of size observed (bp)	Expected size (bp)	No of alleles scored	G”st
1	1	mEgCIR0802	230–272	250	8	0.493
2	2	mEgCIR3282	232–272	245	12	0.310
3	3	mEgCIR0173	122–154	132	5	0.321
4	5	mEgCIR3691	160–260	181	6	0.008
5	6	mEgCIR3543	200–260	232	6	0.006
6	7	mEgCIR2387	250–314	243	13	0.465
7	8	mEgCIR3363	175–228	195	6	0.005
8	9	mEgCIR3886	184–218	187	7	0.006
9	11	mEgCIR3362	130–180	151	7	0.057
10	12	mEgCIR1730	260–290	269	11	0.509
11	13	mEgCIR0832	145–240	240	3	0.032
12	14	mEgCIR3546	286–336	286	11	0.042
13	15	mEgCIR3292	140–212	173	6	0.008
14	16	mEgCIR0353	50–110	102	8	0.812
		Multilocus			109	0.339

G”st - Hedrick's standardized Gst, corrected when population size is small

### Genetic diversity parameters

Fourteen microsatellite markers generated 109 scorable alleles across 532 *E. oleifera* palms. The number of alleles observed ranged from 3 (mEgCIR0832) to 13 (mEgCIR2387) (Table 1). The G”st value for each marker ranged from 0.005 to 0.812. Six markers namely, mEgCIR0802, mEgCIR3282, mEgCIR0173, mEgCIR2387, mEgCIR1730 and mEgCIR0353 revealed high G”st values (>0.250) indicating reasonable power for population discrimination.

The fundamental genetic diversity parameters for each population are summarized in Table 2. The mean number of different alleles (N_a_) was 3.0. The palms originating from K14 recorded highest N_a_ (4.4). The lowest N_a_ (1.8) was exhibited by population C1, located at the other end of the population distribution. H2 recorded the least Shannon’s Information Index, I, (0.220) whereas population K14 revealed the highest (0.602). C6 showed highest observed heterozygosity (H_o_ = 0.218), while H2 exhibited the lowest (0.053) with an average of 0.141. H2 also recorded lowest unbiased H_e_ (H_e_ = 0.112) while K14 showed the highest (0.293) with overall mean of 0.221. The observed heterozygosity values were generally lower than the expected which lead to the positive fixation index (F) for most populations, except for population C6. F values ranged between -0.030 (population C6) and 0.587 (population K2). At the country level, Costa Rica showed higher values, whereas Honduras had the least for most of the genetic diversity parameters.

**Table 2:** Genetic diversity parameters for the 19 *E. oleifera* populations

Country	Population Code	N	N_a_	N_e_	I	Na (rar)	H_o_	H_e_	F
Colombia	C1	30	1.8 (0.261)	1.3 (0.131)	0.252 (0.094)	1.6816	0.119 (0.053)	0.150 (0.057)	0.242 (0.091)
	C5	29	2.4 (0.498)	1.5 (0.199)	0.426 (0.138)	2.0689	0.204 (0.065)	0.239 (0.073)	0.078 (0.084)
	C6	30	2.4 (0.414)	1.4 (0.126)	0.386 (0.109)	2.1300	0.218 (0.062)	0.219 (0.061)	-0.030 (0.045)
	C8	30	2.4 (0.465)	1.5 (0.188)	0.420 (0.131)	2.1668	0.169 (0.053)	0.237 (0.073)	0.240 (0.060)
	C9	29	2.4 (0.359)	1.4 (0.154)	0.401 (0.105)	2.1625	0.197 (0.056)	0.229 (0.060)	0.187 (0.104)
	Mean		2.4 (0.180)	1.4 (0.071)	0.377 (0.051)	2.2004	0.181 (0.026)	0.215 (0.029)	0.141 (0.036)
Panama	P3	30	2.5 (0.327)	1.3 (0.084)	0.335 (0.078)	2.0213	0.128 (0.038)	0.188 (0.047)	0.227 (0.103)
	P5	27	3.9 (0.501)	1.5 (0.173)	0.501 (0.111)	2.9892	0.117 (0.037)	0.250 (0.059)	0.527 (0.062)
	P8	29	2.6 (0.341)	1.2 (0.064)	0.281 (0.075)	2.1863	0.062 (0.017)	0.137 (0.038)	0.289 (0.136)
	P10	30	2.2 (0.434)	1.4 (0.171)	0.361 (0.126)	2.0774	0.139 (0.050)	0.198 (0.066)	0.254 (0.074)
	P12	26	2.7 (0.438)	1.5 (0.186)	0.440 (0.127)	2.4031	0.169 (0.053)	0.239 (0.069)	0.190 (0.066)
	P13	30	2.1 (0.385)	1.4 (0.141)	0.327 (0.112)	1.8635	0.095 (0.045)	0.189 (0.063)	0.388 (0.137)
	Mean		2.7 (0.174)	1.4 (0.058)	0.374 (0.043)	2.4927	0.118 (0.017)	0.200 (0.024)	0.317 (0.041)
Costa Rica	K2	28	3.4 (0.35)	1.2 (0.07)	0.329 (0.052)	2.8601	0.056 (0.010)	0.151 (0.027)	0.587 (0.058)
	K4	30	4.3 (0.304)	1.4 (0.108)	0.557 (0.083)	3.3201	0.126 (0.036)	0.268 (0.044)	0.565 (0.065)
	K8	22	2.1 (0.345)	1.4 (0.137)	0.356 (0.111)	2.0403	0.130 (0.055)	0.199 (0.063)	0.479 (0.120)
	K14	29	4.4 (0.374)	1.5 (0.092)	0.602 (0.074)	3.4944	0.206 (0.028)	0.293 (0.040)	0.259 (0.056)
	K15	30	3.6 (0.355)	1.5 (0.150)	0.475 (0.098)	3.6962	0.150 (0.043)	0.248 (0.054)	0.477 (0.049)
	K21	29	2.3 (0.496)	1.4 (0.152)	0.359 (0.117)	2.0293	0.116 (0.042)	0.221 (0.067)	0.459 (0.063)
	Mean		3.3 (0.172)	1.4 (0.050)	0.449 (0.039)	3.0048	0.130 (0.016)	0.230 (0.022)	0.476 (0.030)
Honduras	H2	30	2.1 (0.327)	1.1 (0.042)	0.220 (0.051)	1.8588	0.053 (0.013)	0.112 (0.027)	0.375 (0.057)
	H3	22	2.1 (0.339)	1.4 (0.112)	0.344 (0.077)	2.0827	0.068 (0.020)	0.194 (0.042)	0.510 (0.034)
	Mean		2.1 (0.204)	1.2 (0.063)	0.282 (0.057)	2.0033	0.060 (0.015)	0.153 (0.032)	0.443 (0.068)
	Overall mean		3.0(0.103)	1.4(0.031)	0.427(0.024)	2.502	0.141(0.010)	0.221(0.013)	0.365(0.020)

N – number of samples in population; N_a_ – number of different alleles; N_e_– number of effective alleles; I = Shannon's Information Index, Na(rar) – allelic richness; H_o_ – observed heterozygosity; H_e_ – unbiased expected heterozygosity; F – fixation index

**Fig. 1:**
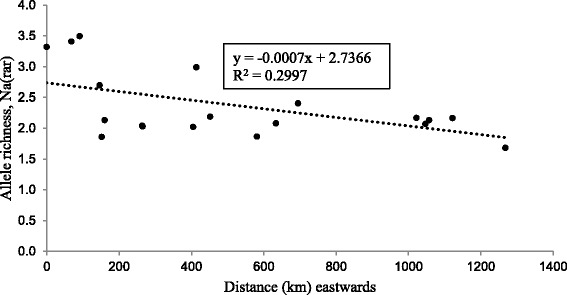
Plot of allelic richness Na(rar) for each population against the position of the populations arranged on a west-to-east direction. Population at the most west (K4) was used to estimate the distances between the populations

The plot of allelic richness against the positions of the 19 populations revealed a decreasing trend of Na(rar) towards the east of the populations’ distribution (Fig. 1). The linear regression analysis further confirmed this result (*p* < 0.05). Approximately, 29.9% of the Na(rar) variation can be explained by the distances in the linear regression.

### Genetic structure and population differentiation

**Fig. 2:**
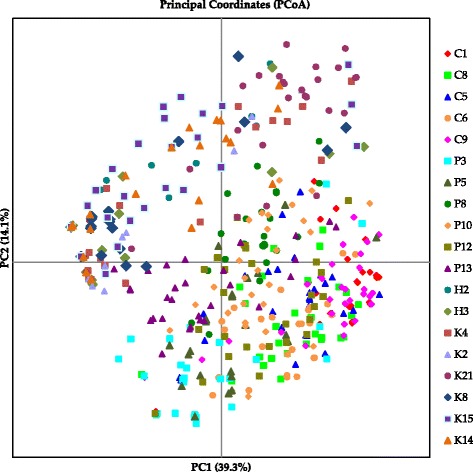
The principle coordinate analysis (PCA) plot of the 532 *Elaeis oleifera* accessions based on Nei’s (1972) genetic distance. The number is parentheses refer to the proportion of variance explained by the principle coordinate

**Fig. 3:**
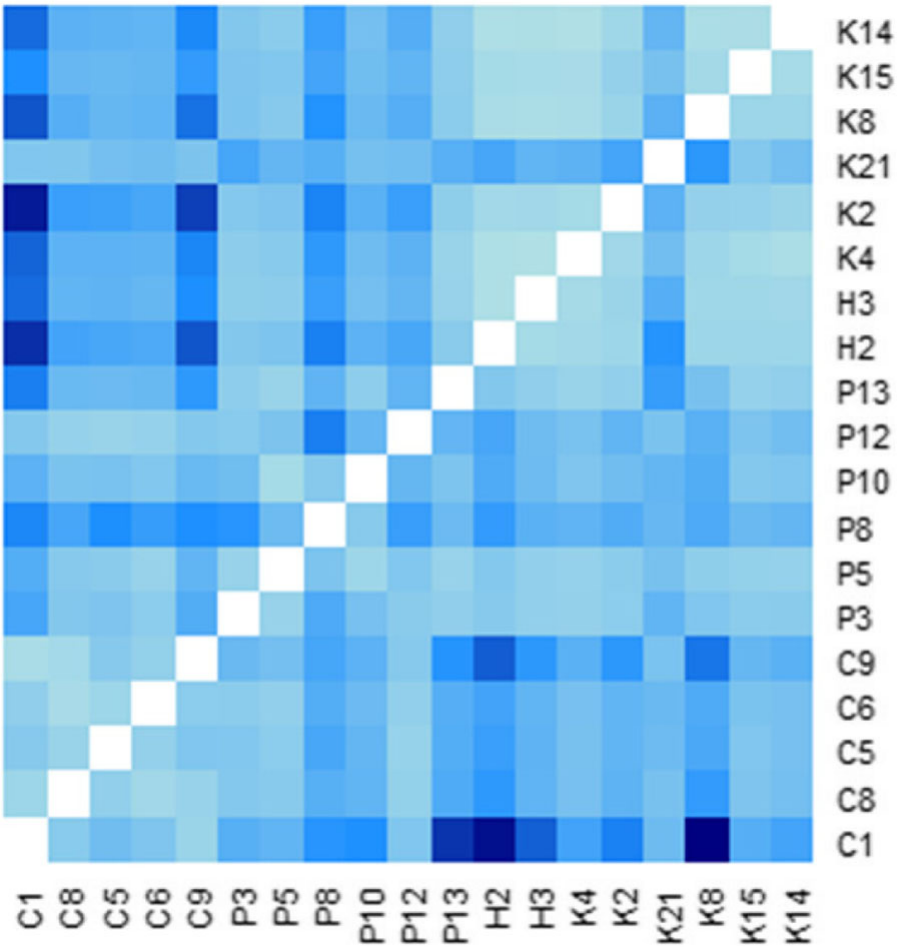
Heatmap illustrating pairwise Fst values among 19 populations. Darker colour indicates stronger pairwise genetic differentiation

The PCoA diagram revealed a single big cluster that consisted of palms from all countries (Fig. 2). Palms from Honduras are dispersed within the distribution area of Costa Rica palms. Some of the palms from Colombia overlapped with those from Panama. The first and second principle component respectively explained 39.3% and 14.1% of the molecular variance. Results from AMOVA showed high and significant overall variation among population (0.290) (Table 3). Pairwise Fst values among populations were in the range of 0.008–0.338 (Table 4 – above diagonal). A heatmap was prepared using these values to provide a more comprehensive overview of the genetic differentiation pattern (Fig. 3 – below diagonal). Population C1 was highly differentiated from four populations namely, H2, H3, P13 and K8 with Fst values above 0.250. Similarly, population C9 also revealed strong genetic differentiation against H2. Twenty-three pairwise Fst values (between 0.150 and 0.250) indicated moderate differentiation, between populations from Colombia and those from Panama, Honduras and Costa Rica. The remaining pairwise Fst values were <0.150 signifying low differentiation between the populations.

**Table 3:** Results from AMOVA indicating partitioning of variation between and within the population

Source	df	SS	MS	Est. Var.	Percent
Among Pops	18	1200.000	66.667	2.191	29%
Within Pops	513	2750.575	5.362	5.362	71%
Total	531	3950.575		7.552	100%

**Table 4:** Pairwise Fst (above diagonal) and genetic distance (below diagonal) across the 19 populations

	C1	C8	C5	C6	C9	P3	P5	P8	P10	P12	P13	H2	H3	K4	K2	K21	K8	K15	K14
C1	0.000	0.060	0.097	0.076	0.034	0.135	0.121	0.191	0.199	0.073	0.291	0.324	0.251	0.164	0.215	0.104	0.338	0.139	0.163
C8	0.028	0.000	0.048	0.024	0.038	0.066	0.062	0.135	0.122	0.045	0.148	0.184	0.123	0.088	0.126	0.087	0.180	0.078	0.092
C5	0.063	0.035	0.000	0.043	0.073	0.070	0.055	0.158	0.118	0.040	0.142	0.173	0.125	0.087	0.120	0.104	0.156	0.073	0.088
C6	0.050	0.013	0.029	0.000	0.055	0.057	0.046	0.141	0.105	0.048	0.139	0.164	0.122	0.088	0.121	0.108	0.147	0.083	0.089
C9	0.011	0.020	0.063	0.045	0.000	0.108	0.092	0.160	0.130	0.064	0.192	0.252	0.185	0.133	0.185	0.084	0.226	0.116	0.133
P3	0.159	0.069	0.078	0.056	0.143	0.000	0.039	0.152	0.089	0.058	0.050	0.065	0.047	0.043	0.055	0.120	0.073	0.057	0.058
P5	0.142	0.065	0.059	0.039	0.121	0.039	0.000	0.080	0.028	0.070	0.038	0.068	0.046	0.042	0.057	0.087	0.051	0.045	0.042
P8	0.210	0.159	0.200	0.176	0.201	0.191	0.104	0.000	0.061	0.174	0.105	0.179	0.133	0.123	0.145	0.115	0.152	0.108	0.118
P10	0.129	0.082	0.085	0.070	0.110	0.099	0.018	0.064	0.000	0.116	0.079	0.147	0.104	0.076	0.098	0.117	0.143	0.068	0.074
P12	0.066	0.041	0.037	0.041	0.067	0.060	0.081	0.217	0.113	0.000	0.119	0.159	0.101	0.086	0.121	0.083	0.132	0.079	0.094
P13	0.217	0.110	0.106	0.111	0.184	0.052	0.035	0.123	0.053	0.120	0.000	0.069	0.049	0.037	0.046	0.175	0.087	0.042	0.048
H2	0.296	0.164	0.157	0.151	0.260	0.069	0.080	0.216	0.130	0.158	0.058	0.000	0.018	0.022	0.028	0.192	0.030	0.030	0.028
H3	0.239	0.121	0.125	0.116	0.202	0.051	0.054	0.171	0.093	0.117	0.038	0.003	0.000	0.016	0.032	0.139	0.025	0.025	0.024
K4	0.247	0.129	0.129	0.126	0.211	0.054	0.062	0.183	0.101	0.128	0.036	0.002	0.006	0.000	0.024	0.094	0.029	0.018	0.008
K2	0.315	0.172	0.167	0.158	0.280	0.066	0.078	0.212	0.132	0.171	0.051	0.022	0.023	0.018	0.000	0.129	0.043	0.042	0.033
K21	0.073	0.072	0.092	0.097	0.081	0.160	0.117	0.135	0.090	0.094	0.134	0.159	0.122	0.128	0.161	0.000	0.186	0.067	0.091
K8	0.260	0.139	0.116	0.121	0.231	0.079	0.063	0.195	0.109	0.138	0.057	0.011	0.009	0.012	0.034	0.131	0.000	0.029	0.029
K15	0.201	0.114	0.112	0.116	0.180	0.078	0.070	0.162	0.099	0.121	0.053	0.017	0.015	0.013	0.043	0.087	0.019	0.000	0.015
K14	0.239	0.131	0.127	0.120	0.208	0.069	0.057	0.173	0.092	0.140	0.049	0.004	0.005	0.000	0.025	0.118	0.009	0.007	0.000

**Fig. 4:**
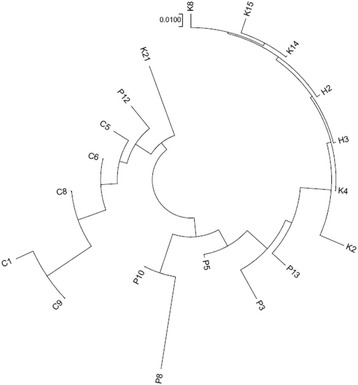
Phylogenetic tree using Nei’s genetic distance estimates

The genetic distance among the population was highest (0.315) between C1 and K2 while the lowest was 0.003, between H2 and H3 (Table 4 – below diagonal). The phylogenetic tree constructed according to these estimates revealed two main groups: populations from Costa Rica, Panama and Honduras formed one group while, populations from Colombia established another cluster, together with P8 and K21 (Fig. 4).

**Fig. 5:**
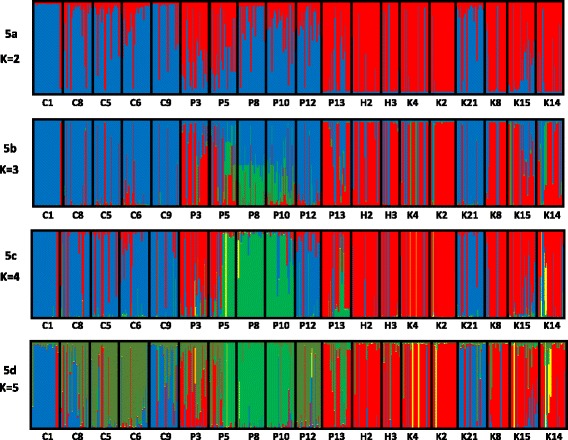
Illustration of results from Structure analyses. Graphs 5a to 5d represents boxplot for K = 2, 3, 4 and 5 respectively

The LnP(D) result from Structure v2.2 showed gradual increment from K = 1 to K = 10 without clear peak (Additional file 1). Thus, we further examined the results attained for K = 2, 3, 4 and 5 (Fig. 5a, b, c and d). For K = 2, populations from Honduras, Costa Rica (except K21) and three Panama populations (P3, P5 and P13) formed one subpopulation whereas the remaining populations (mainly from Colombia, together with P8, P10, P12 and K21) created the second subpopulation. At K = 3, three subgroups were attained due to the separation of P8 and P10 from the second subpopulation. Three subgroups were also retained for K = 4. At K = 5, the populations in subpopulation one broke up into two groups; populations C1, C9 and K21 differentiated from C5, C6, C8 and P12. Populations P8 and P10 remain distinct at K = 5.

The results from PowerCore are presented in Table 5. A total of 34 palms were shortlisted. These include 7 palms from Colombia, 10 from Panama, 13 from Costa Rica and another 4 from Honduras.

**Table 5:** Results from PowerCore indicating the number of palms that collectively reduced redundancy and maximized diversity for establishment of core collection

Country	Total number of palms analysed	Number of palms proposed for core collection
Colombia	148	7
Panama	172	10
Costa Rica	168	13
Honduras	52	4
Total	532	34

## Discussion

### Cross transferability

In this study, 77.7% of the *E. guineensis* microsatellites recorded successful cross-amplification in *E. oleifera*. Successful cross-species amplification of SSRs developed from genomic information of *E. oleifera* [19] and *E. guineensis* [20] have been reported previously. Cross-species amplification of SSRs has also been reported in many plant species for instance napier grass [21], Eucalyptus [22], *Jathropa* [23], *Rhododendron* [24], *Lavandula* species [25], sugarcane [26] and *Dendrobium* [27]. The average transferability rate of SSRs among species within the same genus was reported at approximately 73% [28], a figure that is comparable to the value attained in this study. Mating system and life span are among the factors influencing cross amplification of SSRs. Oil palm is an out crossing species with long generation time. Such species is expected to record higher SSR transferability than selfed- and short-lived species [28]. Therefore, the high cross amplification rate attained in the current work is expected.

The high transferability rate of SSRs among the *Elaeis* species and the comparable size of the amplified products attained in the *oleiferas* samples could also be the result from the highly conserved sequences flanking the microsatellite regions. Moreover, the success of SSR locus amplification across-species is higher when genetic distance between the species is small. This was demonstrated in a phylogenetic analysis carried out based on annotated subset of proteins from *E. guineensis* and *E. oleifera*, where the two species revealed close genetic relationship [29]. Furthermore, there is also evidence on the successful application of *E. guineensis* SSRs for constructing the genetic linkage map involving oil palm interspecific hybrids (*oleifera* x *guineensis*) [17] as well as their backcrosses [30].

However, a number of SSRs (34%) failed to generate amplified product in *E. oleifera*. Mutation has been reported as one of the possible causes of failure in SSR amplification [31]. Mutation that occurs at the SSR primer binding sites may prevent annealing and subsequently result in no amplification of PCR products [32]. These can include indels or nucleotide substitutions in the primer binding sites [33].

### Genetic diversity of the *E. oleifera*

Previous studies on evaluating *E. oleifera* genetic materials by means of molecular markers are quite limited. [34] analyzed natural populations collected from the Amazon forest in Brazil using ninety-six Random Amplified Polymorphic DNA (RAPD) markers. The group reported moderate level of diversity compared to *E. guineensis* accessions. The group also found that the palms were clustered according to their distribution along the Amazon River rather than geographic distances. The river network provided means for seeds dispersal. Therefore, palms along the river were grouped together.

A primary attempt to develop simple sequence repeats (SSRs) from DNA extracted from selected *E. oleifera* was reported in 2010 [35]. Successful amplification of the *oleifera* SSRs was attained across DNA of *E. guineensis, Cocos nucifera* and *Jessinia bataua* [19]. Further, marker data analysis revealed considerable level of genetic diversity among the populations and clear grouping of samples according to the species.

Recently, [36] described the genetic diversity of *Elaeis oleifera* natural populations assembled from four countries, namely Peru, Brazil, Colombia and Ecuador as well as two hybrid populations created from *E. oleifera* and *E. guineensis*. Using 13 SSRs developed by [14], four genetic groups could be distinguished. These groups corresponded with the country where the accessions were assembled. The two hybrid populations were clustered respectively into Colombia and Brazil groups, in agreement with the origin of the female parents used to create them. Significant differences between countries were reported for several phenotypic data such as mesocarp-to-fruit and oil-to-bunch ratios. Further analysis of both, the molecular and phenotypic data revealed the number of entries per country that are needed in the core collection for long term conservation.

In this study, we have determined the genetic diversity of 19 *E. oleifera* populations using 14 *E. guineensis* SSRs. Previously, [15] and [36] had applied 16 and 13 SSRs respectively across populations of *E. guineensis* and *E. oleifera* with satisfactory results. The average number of alleles per locus and expected heterozygosity in *E. oleifera* populations (Na = 3.00; He = 0.221) analysed in this study were lower than that detected in *E. guineensis* (Ao = 5.0; He = 0.644) [15], indicating a rather low genetic diversity of the American oil palm populations. Similar observation was also reported by [19, 35] in a study carried out on small number of *E. guineensis* and *E. oleifera* samples. The *oleifera* populations were reportedly less diverse too, in terms of the phenotypic traits especially palm height, mesocarp ratio and nut weight [6]. Nevertheless, *E. oleifera* offers genes that are not available in the *guineensis*. Genes for low height increment, unsaturated fatty acids and high carotene content [6, 8] can be exploited for improvement of the *E. guineensis.*

At the population level, one of the populations from Colombia (C6) possessed negative fixation index (F) value (-0.030). This population was located at Cerete, Colombia. Bulk of the natural groves of *oleiferas* in Cerete was removed to give way to agriculture [5]. The remaining genetic materials were therefore very sparse and located far from each other. In addition, during the exploration for genetic materials, the collection team noted the presence of two oil palm mills in this area [5] indicating extensive exploitation of the *oleifera* fruits by the locals. *Oleifera* bunches may have been transported from far for oil extraction at these mills. It has been shown that oil palm seeds from the processed bunches preserve germination ability thus, unrelated palms from these bunches could have been established in areas near the mills in Cerete. Hence, the genetic materials assembled from this area possess dissimilar genotypes, introduced by the unrelated palms, which is reflected in higher H_o_ value. This leads to the negative F value, as seen for the population from Cerete (C6).

Allelic richness is a useful estimate for evaluating population diversity. The plot of allelic richness Na(rar) of each population against position (Fig. 1) indicated significant decreasing trend of allele richness from Costa Rica towards two countries namely, Colombia and Honduras. High genetic diversity estimates were also attained for the populations from Costa Rica. These findings suggest that the country can be denoted as the center of diversity for *E. oleifera*. Analysis carried out on the *E. oleifera* remains across an area between the southern part of the United States and the south of Uruguay [37] concluded that the establishment of the *E. oleifera* populations in Colombia was more recent. This archaeological evidence further supports the low Na(rar) and genetic diversity among the Colombia populations. Similarly, populations from Honduras also exhibited low diversity. However, the low diversity observed among the genetic materials from Honduras is probably due to founder effect. Here, we only investigated small number of populations ie. two from this country thus, limited diversity was captured.

### Population structure and differentiation

The AMOVA revealed that 29% of the variation observed in this study was attributed by the genetic differentiation between the populations. The differences between the populations are considered extensive, thus sampling a limited number of individuals from many populations should be implemented for future collection and establishment of core collection. Our results are in agreement with that reported by [5], based on phenotypic variation. The genetic differentiation between populations in *E. oleifera* is higher than that reported in *E. guineensis* (0.206) [15]. Extensive distribution of *E. guineensis* wild groves was reported along the oil palm belt stretching from the west to the central Africa. However, unlike *E. guineensis*, the natural distribution of *E. oleifera* was highly discontinuous [5]. In such condition, mating involving individual within a population was more common. Thus, higher differentiation among population is expected in this study.

The phylogenetic tree presented in Fig. 4 indicated grouping of the populations according to country except for P12 (from Panama) and K21 (from Costa Rica). Similar results are also observed in Fig. 5b and c. In these figures, P12 and K21 revealed different genetic attributes as compared to other populations from Panama and Costa Rica, respectively. The results presented in Fig. 5 indicated that gene flow generally occurred at different rate among individuals across populations and countries thus, no populations exhibited absolute uniformity. Gene flow was clearly evidenced across countries, particularly between P12, K21 and the Colombian populations as well as between populations from Honduras and Costa Rica. At K = 5 (Fig. 5d), P12 revealed almost similar genetic attributes with C5 and C6. P12 is the nearest collection site to Colombia, located at the east of the Panama Canal. Considerable rate of genetic exchange could have occurred among these populations which, explains their grouping.

Between 1967 and 1976, a private company in Costa Rica initiated collections of *E. oleifera* seeds from natural populations in Panama, Colombia, Suriname, Honduras, Nicaragua and Brazil [38]. These genetic materials were planted in Coto for phenotypic evaluation. One of the populations analysed in this study was also assembled from Coto (K21). Our results showed that K21 is genetically similar to populations C1 and C9 from Colombia (Fig. 5d). The individual palms collected from population K21 could be the result from hybridization between palms naturally found in Costa Rica and those assembled from Colombia [38]. This possibly explained the close relationship of K21 with the Colombian populations.

### Implication for breeding and future conservation programs

The current *ex situ* living collection of *E. oleifera* occupies approximately 18 hectares of land, accommodating approximately 2,500 palms. Maintaining living collection for large crop like oil palm is expensive. Nonetheless, field evaluation of the collection revealed some interesting traits useful for oil palm genetic improvement [6]. Achieving genetic gain through conventional breeding takes a very long time for oil palm. This is because approximately ten years is required to complete one cycle of field data collection and evaluation for the species. Therefore, appropriate planning to preserve individual palms that possess traits of interest should be in place to ensure long-term accessibility. The principles of core collection where maximum genetic variation is preserved in a reduced land size can be applied. The establishment of such collection is an ideal option for oil palm as it increases efficiency of conservation and allows for a more effective access to the genetic materials.

The results presented above facilitate the identification of unique populations or rich allelic individual palms as well as populations that exhibit high genetic variation. These results are useful for selecting genetic materials for establishing the core collection. Some genetically variable populations were identified based on the genetic diversity parameters estimated in this study. Among the interesting populations are C5, C8, P5, P12, K4, K14 and K15, as they exhibited genetic diversity estimates higher than the mean. The results from AMOVA indicated greater diversity between populations which suggests that sampling limited individuals from many populations should capture maximum diversity. Previous analysis [39] revealed that analyzing population size of 20 and 30 individuals resulted in comparable genetic diversity measures in oil palm. Therefore, preserving 20–30 seeds per population may cover the genetic diversity present in the population. In addition, 34 rich allelic palms were identified from PowerCore analyses. Offsprings could then be created through selfing or intercrossing of these palms. Efforts to preserve these materials further should be initiated to ensure selection gains and accessibility in the future.

Our results also revealed high pairwise genetic differentiation (Fst) between populations from Colombia and those from other countries. This can be visualized in Fig. 3 - below diagonal. Crossing program can be initiated using palms from two populations that revealed dark blue for instance, C1 and K8, C1 and H2, C1 and H3, C1 and P13 or C9 and H2. Based on the phenotypic data available [6, 8, 10], individual palms exhibiting interesting traits can be selected from each of these populations. Palms that possess low height increment, high mesocarp content, high carotene content and high oil unsaturation can be included in the crossing scheme. Such breeding programme can achieve two goals, first, incorporating the traits of interest into the next generation for future access and secondly, retaining the overall genetic variability for selection gains in the future.

## Conclusion

From the current work, 77% of the *E. guineensis* SSRs tested showed cross amplification in *E. oleifera*. Of these, 40% showed discriminative power among populations thus, proving to be a reasonably useful marker resources for genetic studies of *E. oleifera*. The genetic diversity estimates indicated high genetic variability among the populations from Costa Rica. This result suggests that the country may serve as the centre of diversity of *E. oleifera*. This study also provides valuable information that help oil palm breeders and germplasm manager in identifying individual palms and populations for establishment of core collection. Implementing the suggestions described above would result in comprehensive genetic collection that retains genetic diversity as well as valuable traits for selection gains and access in the future.

## Methods

### Plant materials and DNA extraction

**Fig. 6:**
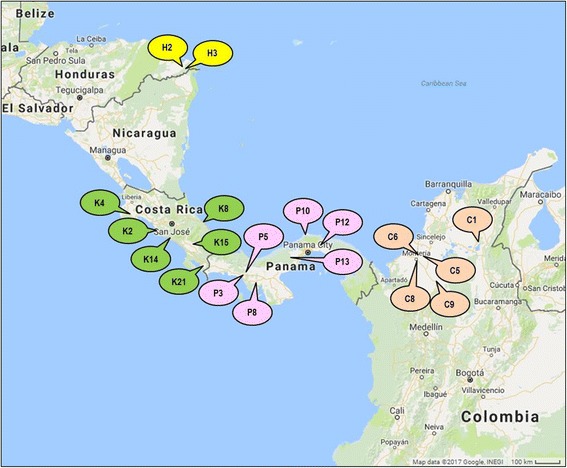
Map showing the distribution of the *E. oleifera* populations in Honduras, Costa Rica, Panama and Colombia that were included in the study

A total of 532 palms representing 19 populations were sampled from the MPOB *ex situ* living collection of *E. oleifera*. These populations were selected based on their geographic distribution, microclimates, altitudes and rainfalls in Colombia, Panama, Costa Rica and Honduras (Additional file 2). For Colombia, we included genetic materials collected from a wider range of areas as compared to that reported by [36]. Figure 6 indicates the distribution of the populations analysed in this study. For each population, 22–30 palms were sampled depending on availability of the materials. Young unopened leaves were harvested from each palm, cleaned, and kept in -80 °C.

Genomic DNA was extracted from 3 g of fresh leaf using CTAB DNA extraction [40] with minor modification. The DNA samples were quantified by optical density (OD) reading using spectrophotometer (UV/VIS Spectrometer Lambda Bio, Perkin Elmer, USA). The DNA purity was further tested with DNA digestion technique using two restriction enzymes namely, EcoRI and HaeIII, followed by separation on 1.0% agarose gel in TBE buffet at 100 V for 1 h. The DNA was visualized after ethidium bromide staining under ultraviolet exposure.

### Testing for Transferability of *E. guineensis* microsatellite markers to *E. oleifera*

In this study, 18 microsatellite markers designed based on [14], were tested for their polymorphism in *E. oleifera*. A screening panel that contained three DNA samples of each population was established for testing the markers. The details of the 18 microsatellite markers applied are indicated in Additional file 3. These markers were selected to represent each linkage group based on the genetic map constructed by [14].

### Microsatellite amplification

The total volume of each PCR reaction was 12.5 μl, comprising 1x PCR buffer (50 mM KCl, 10 mM Tris-HCl (pH 9.1 at 21 °C), 0.1% TritonTMX-100), 1.5 mM Mg2Cl, 200 μM dNTPs mix, 1.0 U Taq DNA polymerase (Vivantis, Malaysia), 0.25 μM of each primer, 0.38 μM fluorescent dye (Applied Biosystems, USA) and 50 ng of DNA template. The PCR conditions included initial denaturation at 94 °C for 5 min, followed by 35 cycles of denaturation at 94 °C for 30 s, annealing at 52 °C for 1 min and elongation at 72 °C for 2 min, then final elongation step of 72 °C for 10 min. The M13-tailed forward primer was labeled with 4-colour fluorescent dyes. The fluorescent dyes used in the study were FAM (blue), PET (red), VIC (green) and NED (yellow). The sample set was subdivided into four panels and each of them consisted of four microsatellite primers labeled with four fluorescent dyes, respectively.

### Fragment analysis and genotyping

Prior to fragment analysis, PCR products from four different SSR primers were combined at 1:1:1:1 ratio. The multiplex (2.0 μl) was pretreated by adding 7.80 μl of Hi-DiTM Formamide (Applied Biosystems, USA) and 0.20 μl of GeneScanTM-500 LIZTM Size Standard (Applied Biosystems, UK) to make up 10.0 μl of the final volume. The mixture was vortexed thoroughly and followed by denaturation at 95 °C for 5 min. The plate was then placed on the ice immediately for another 5 min before capillary electrophoresis using ABI PRISM® 3100 Genetic Analyzer (Applied Biosystems, USA). The raw reads were retrieved and exported to GeneMapper V3.1 software. The genotype profiles were finally generated and displayed in the software for scoring purpose.

### Data analysis

Marker informativeness was evaluated based on the G”st values determined in GenAlex version 6.5 [41, 42]. These estimates were computed after correction for small population size based on [43] and [44] as described in [41, 42]. In addition, the fundamental genetic diversity parameters such as allele frequencies, average number of different allele (N_a_), number of effective alleles (N_e_), Shannon Information Index (I), observed heterozygosity (H_o_), unbiased expected heterozygosity (H_e_) and fixation index (F) were also estimated for each population.

Allelic richness (Na (rar)) for each population was estimated using HP-Rare 1.1 software [45, 46]. Generally, large population size possesses more alleles. In HP-Rare 1.1 software, allelic richness is estimated in a standardized sample size for each population which, results in more accurate valuation. The allelic richness results were plotted against the distance between the populations. The distance was estimated using the longitude information of each population against the longitude of the population located at the most west, that is K4 (population 4 of Costa Rica). Distances (in kilometers) were computed based on the longitude values using a calculator available at the USA National Hurricane Centre (http://www.nhc.noaa.gov/gccalc.shtml).

GenAlex version 6.5 was also applied in determining the genetic relatedness among the individual palm in the form of Principle Component Analysis (PCoA) plot. AMOVA was carried out to generate the overall partitioning of the genetic variation within and between the populations. Fst values and genetic distances (GD) [47] were estimated to provide further detailed information of relatedness among the populations. Phylogenetic tree was later constructed in ITOL website (http://itol.embl.de/upload.cgi) [48] using the tree scripts attained from the GD values. The pairwise GD and Fst estimates among the sampled populations were further illustrated in a heatmap using R scripts.

Structure V2.2 program [49] was applied to assign the palms into subpopulations. Analysis was carried out with the assumption that the number of groups or admixtures (K) was unknown. The parameters were set at burn-in of 10,000 with 100,000 repeats for K values ranging from 1–10.

We applied PowerCore software [50] to shortlist individual palms that collectively represent optimum diversity and reduced duplication of the *E. oleifera* analyzed in this study. The software was developed using a heuristic algorithm that retained all different alleles and the results were highly reproducible.

## Additional files

Additional file 1: Log probability data (LnP(D)) as function of k (number of clusters) from the STRUCTURE. (DOCX 2712 kb)

Additional file 2: Information of country, collection sites and population size included in the study. (DOCX 16 kb)

Additional file 3: Details of the 18 microsatellite markers used in the study. (DOCX 16 kb)

## Abbreviations

AMOVA: analysis of molecular variation
CRD: Completely randomized design
CTAB: cetyltrimethylammonium bromide
iTOL: interactive tree of life
MPOB: Malaysian palm oil board
PCoA: Principle component analysis
PCR: Polymerase chain reaction
RAPD: Random amplified polymorphic DNA
SSRs: Simple sequence repeats
