# Supplementary material for: Genetic diversity of Elaeis oleifera (HBK) Cortes populations using cross species SSRs: implication’s for germplasm utilization and conservation
Source: BMC Genet. 2017 Apr 19;18:37. doi: 10.1186/s12863-017-0505-7 (PMC5395919; doi:10.1186/s12863-017-0505-7)
Supplement: Supplementary file 1 — Log probability data (LnP(D)) as function of k (number of clusters) from the STRUCTURE. (DOCX 2712 kb) [file 12863_2017_505_MOESM1_ESM.docx]

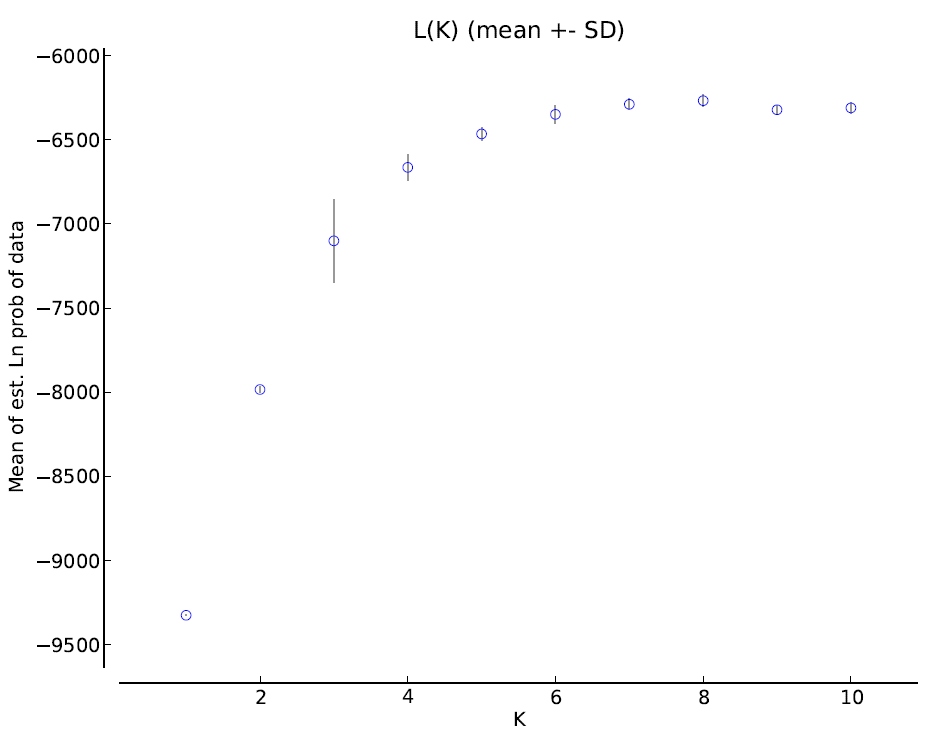


**Additional file 1: Log probability data (LnP(D)) as function of k (number of clusters) from the STRUCTURE**
