# Supplementary material for: Genetic diversity of Elaeis oleifera (HBK) Cortes populations using cross species SSRs: implication’s for germplasm utilization and conservation
Source: BMC Genet. 2017 Apr 19;18:37. doi: 10.1186/s12863-017-0505-7 (PMC5395919; doi:10.1186/s12863-017-0505-7)
Supplement: Supplementary file 2 — Information of country, collection sites and population size included in the study. (DOCX 16 kb) [file 12863_2017_505_MOESM2_ESM.docx]

**Additional file 2: Information of country, collection sites and population size included in the study**

| **Country** | **Collection site** | **Population code** | **No of palms sampled** | **Latitude north** | **Longitude west** |
| --- | --- | --- | --- | --- | --- |
| Colombia | Santa Halena, Chimichagua | C1 | 30 | 9.4264 | -73.8601 |
|  | Monteria | C5 | 29 | 8.75098 | -75.8785 |
|  | Cerete | C6 | 30 | 8.8888 | -75.7896 |
|  | La Victoria, Monteria | C8 | 30 | 8.6047 | -76.1099 |
|  | Caucasia | C9 | 29 | 7.9797 | -75.1986 |
|  |  |  |  |  |  |
| Panama | 3km from Guabala junction | P3 | 30 | 8.2222 | -81.7506 |
|  | 11km from Guabala junction | P5 | 27 | 8.1701 | -81.6698 |
|  | Sona | P8 | 29 | 8.0250 | -81.3255 |
|  | Puertobelo | P10 | 30 | 9.5489 | -79.6530 |
|  | Chepo | P12 | 26 | 9.1632 | -79.1040 |
|  | Rio Indio (2km from Icacal) | P13 | 30 | 8.7787 | -80.1432 |
|  |  |  |  |  |  |
| Costa Rica | Puntarenas | K2 | 28 | 9.9711 | -84.8315 |
|  | Nicoya | K4 | 30 | 10.1432 | -85.4534 |
|  | Limon | K8 | 22 | 9.9913 | -83.0415 |
|  | Jaco | K14 | 29 | 9.6202 | -84.6217 |
|  | Quepos | K15 | 30 | 9.4295 | -84.1224 |
|  | Coto | K21 | 29 | 8.5357 | -83.0348 |
|  |  |  |  |  |  |
| Honduras | Rio Kruta | H2 | 30 | 14.8908 | -84.0645 |
|  | 15km from Rio Kruta | H3 | 22 | 14.7908 | -83.0645 |
