# Supplementary material for: Genetic diversity of Elaeis oleifera (HBK) Cortes populations using cross species SSRs: implication’s for germplasm utilization and conservation
Source: BMC Genet. 2017 Apr 19;18:37. doi: 10.1186/s12863-017-0505-7 (PMC5395919; doi:10.1186/s12863-017-0505-7)
Supplement: Supplementary file 3 — Details of the 18 microsatellite markers used in the study. (DOCX 16 kb) [file 12863_2017_505_MOESM3_ESM.docx]

**Additional file 3: Details of the 18 microsatellite markers used in the study**

| **No** | **Linkage group** | **Microsatellite loci** | **Motif** | ***Primer Sequences (5'-3')** | **Expected size (bp)** |
| --- | --- | --- | --- | --- | --- |
| 1 | 1 | mEgCIR0802 | (GA)_12_ | M13-CTCCTTTGGCGTATCCTTTA  TACGTGCAGTGGGTTCTTTC | 250 |
| 2 | 2 | mEgCIR3282 | (GA)_20_ | M13-GTAACAGCATCCACACTAAC  GCAGGACAGGAGTAATGAGT | 245 |
| 3 | 3 | mEgCIR0173 | (GA)_18_ | M13-TGAACAAGAAGGCGGAAAGAGA  TGCGGGCGAGGAAAGGT | 132 |
| 4 | 4 | mEgCIR1753 | (GA)21 | M13-GCAGGGATTAAGTTTGATAT | 335 |
| 5 | 5 | mEgCIR3691 | (GA)_14_ | TTTGATGTTGCTTCTTTGAT | 181 |
| 6 | 6 | mEgCIR3543 | (GA)_17_ | M13-GTTCCCTGACCATCTTTGAG  GTCGGCGATTGATTAGATTC | 232 |
| 7 | 7 | mEgCIR2387 | (GA)_11_ | M13-TTGGTGAGCCATTTGCTACA  CCTCCTTCCACCCCTCTACT | 243 |
| 8 | 8 | mEgCIR3363 | (GA)_17_ | M13-CTTGACAATACCCTGAGTAGTAG  GCTGTGCCTATCGGACTT | 195 |
| 9 | 9 | mEgCIR3886 | (GA)_5_GT(GA)_20_ | M13-TTCTAGGGTCTATCAAAGTCATAAG  AGCCACCACCACCATCTACT | 187 |
| 10 | 10 | mEgCIR3785 | (GA)21 | M13-AAGCAATATAGGTTCAGTTC | 284 |
| 11 | 11 | mEgCIR3362 | (GA)_19_ | TCATTTTCTAATTCCAAACAAG |  |
| 12 | 12 | mEgCIR1730 | (GT)_22_ | M13-AATTTCAAATACAGCATAGC  CATAGTAAGTTTTGGATGATTATTA | 269 |
| 13 | 13 | mEgCIR0832 | (GA)_19_ | M13-CTCCGATGGTCAAGTCAGA  AAATGGGGAAGGCAATAGTG | 240 |
| 14 | 14 | mEgCIR3546 | (GA)_15_ | M13-GCCTATCCCCTGAACTATCT  TGCACATACCAGCAACAGAG | 286 |
| 15 | 15 | mEgCIR3292 | (GA)_20_ | M13-AGCCATGAGTGAATCATATC  ACCACGATGTCAATCTCTAT | 173 |
| 16 | 16 | mEgCIR0353 | (GT)_11_(GA)_15_ | M13-ATTTCGTAAGGTGGGTGT  CCTCCAAACTCCTCTGT | 102 |
| 17 | 5 | mEgCIR3574 | (GA)_19_ | M13-AGAGACCCTATTTGCTTGAT  GACAAAGAGCTTGTCACAC | 207 |
| 18 | 7 | mEgCIR3300 | (GA)_19_ | M13-CATGCACGTAAAGAAAGTGT  CCAAATGCACCCTAAGA | 186 |
